# Ribavirin as a potential therapeutic for atypical teratoid/rhabdoid tumors

**DOI:** 10.18632/oncotarget.23883

**Published:** 2018-01-03

**Authors:** Joshua Casaos, Sakibul Huq, Tarik Lott, Raphael Felder, John Choi, Noah Gorelick, Michael Peters, Yuanxuan Xia, Russell Maxwell, Tianna Zhao, Chenchen Ji, Thomas Simon, Julie Sesen, Sarah J. Scotland, Richard E. Kast, Jeffrey Rubens, Eric Raabe, Charles G. Eberhart, Eric M. Jackson, Henry Brem, Betty Tyler, Nicolas Skuli

**Affiliations:** ^1^ Hunterian Neurosurgical Research Laboratory, Neurosurgery Department, Johns Hopkins School of Medicine, Johns Hopkins University, Baltimore, MD 21231, USA; ^2^ Center for Vascular and Inflammatory Diseases, School of Medicine, University of Maryland, Baltimore, MD 21201, USA; ^3^ INSERM U1037, Centre de Recherche en Cancérologie de Toulouse, CRCT, 31100 Toulouse, France; ^4^ IIAIGC Study Center, Burlington, VT 05401, USA; ^5^ Pathology Department, Johns Hopkins School of Medicine, Johns Hopkins University, Baltimore, MD 21231, USA

**Keywords:** ribavirin, atypical teratoid/rhabdoid tumor, glioma, therapy, brain tumors

## Abstract

Atypical teratoid/rhabdoid tumors (AT/RT) are highly aggressive, malignant tumors and are the most common malignant brain tumor in children under 6 months of age. Currently, there is no standard treatment for AT/RT. Recent studies have reported potential anti-tumoral properties of ribavirin, a guanosine analog and anti-viral molecule approved by the Food and Drug Administration for treatment of hepatitis C. We previously demonstrated that ribavirin inhibited glioma cell growth *in vitro* and *in vivo*. Based on these results and the fact that no pre-clinical model of ribavirin in AT/RT exists, we decided to investigate the effect of ribavirin on several human AT/RT cell lines (BT12, BT16, and BT37) both *in vitro* and *in vivo*. We provide evidence that ribavirin has a significant impact on AT/RT cell growth and increases cell cycle arrest and cell death, potentially through modulation of the eIF4E and/or EZH2 pathways. Interestingly, using scratch wound and transwell Boyden chamber assays, we observed that ribavirin also impairs AT/RT cell migration, invasion, and adhesion. Finally, we demonstrate that ribavirin significantly improves the survival of mice orthotopically implanted with BT12 cells. Our work establishes that ribavirin is effective against AT/RT by decreasing tumoral cell growth and dissemination and could represent a new therapeutic option for children with this deadly disease.

## INTRODUCTION

Tumors of the central nervous system (CNS) are the most common solid malignancies in adults and second most common malignancy overall in children. Specifically, pediatric gliomas account for 28% of all pediatric brain tumors, making up 80% of malignant brain tumors according to the Central Brain Tumor Registry of the United States [1]. Atypical teratoid/rhabdoid tumors (AT/RT) are highly aggressive CNS tumors that comprise approximately 1–2% of overall pediatric brain tumors, yet account for 20% of cases in children 3 years old and younger. AT/RT are the most common malignant brain tumors in children under 6 months of age, and their characteristics of aggressive growth, young age at diagnosis, and propensity to disseminate along the neuroaxis all contribute to the poor prognosis that these tumors carry [2, 3]. Overall survival is poor in patients with AT/RT, with reported median survival of approximately 1 year. Importantly, there is no current standard treatment for children diagnosed with AT/RT. While the standard of care for patients with high grade gliomas is typically surgical resection, if possible, followed by radiotherapy and temozolomide (TMZ) [4], the high frequency of AT/RT in patients younger than 3 years old complicates the use of radiation therapy following surgical resection [3]. Additionally, overall response to chemotherapeutic interventions remains discouraging, highlighting the significant need for innovative therapies.

Ribavirin is a guanosine analogue well described for its anti-viral properties and has been used to treat hepatitis C, influenza, subacute sclerosing panencephalitis (SSPE), and respiratory syncytial virus (RSV) [5–7]. The growing cost of medical care, particularly in oncology, has significantly increased the repurposing of existing clinically used drugs to alleviate the financial burden of drug development. Interestingly, we and others recently demonstrated that ribavirin has anti-tumor effects on leukemia [8, 9], glioblastoma (GBM) [10], colon cancer [11] and breast cancer cells [12, 13]. Indeed, it has been shown that ribavirin sensitizes colon cancer cell lines to oxaliplatin [11], reduces breast cancer proliferation/migration/invasion/metastasis, and may target radioresistance in metabolically hyperactive tumors [13]. Our team also highlighted the therapeutic effect of ribavirin on gliomas as we previously demonstrated that ribavirin had some promising anti-cancer properties *in vitro* and most importantly *in vivo* in GBM and GBM stem-like cells [10]. The mechanisms of ribavirin's anti-tumor effects have only recently begun to be elucidated, and the effects are thought to be mediated via multiple pathways, including extracellular regulated protein kinases (ERK) in the mitogen-activated protein kinase (MAPK) pathway [14], eukaryotic translation initiation factor 4E (eIF4E) [15], mitogen-activated protein kinase interacting protein kinase 1 (MNK1) [14], inosine 5′-monophosphate dehydrogenase (IMPDH) [16], and/or enhancer of zeste homolog 2 (EZH2) [17]. EZH2 in particular has a role in transcriptional repression through H3K27 tri-methylation and is considered an attractive epigenetic target for cancer therapy. Intriguingly, a recent landmark study identifying distinct molecular subtypes of AT/RT demonstrated that EZH2 was one of three genes that were highly expressed in almost all AT/RT compared with normal brain tissue [18]. Additionally, other studies suggest that the inhibition of EZH2 may alter cell cycle progression and induce radiation sensitivity in AT/RT [19]. Taken together, these recent findings suggest that ribavirin could potentially be of therapeutic interest in AT/RT.

In the present work, we evaluated the efficacy of ribavirin in treating pediatric AT/RT in three different cell lines (BT12, BT16, and BT37) *in vitro* and *in vivo*. We demonstrated that ribavirin impairs AT/RT cell growth, arrests the cell cycle, and increases AT/RT cell death *in vitro*. Additionally, we observed that ribavirin decreases AT/RT cell migratory, invasive, and adhesive capacities. Mechanistically, we provide evidence that ribavirin treatment modulates known key pathways, such as EZH2, p21, and/or eIF4E. Most importantly, we showed that mice intracranially implanted with BT12 cells and treated with ribavirin lived longer compared to vehicle-treated controls. As no current effective treatments for children diagnosed with AT/RT are available, ribavirin represents an exciting new potential therapeutic for these patients.

## RESULTS

### Ribavirin decreases AT/RT cell proliferation, potentially through cell cycle arrest and induction of cell death

**Figure 1:**
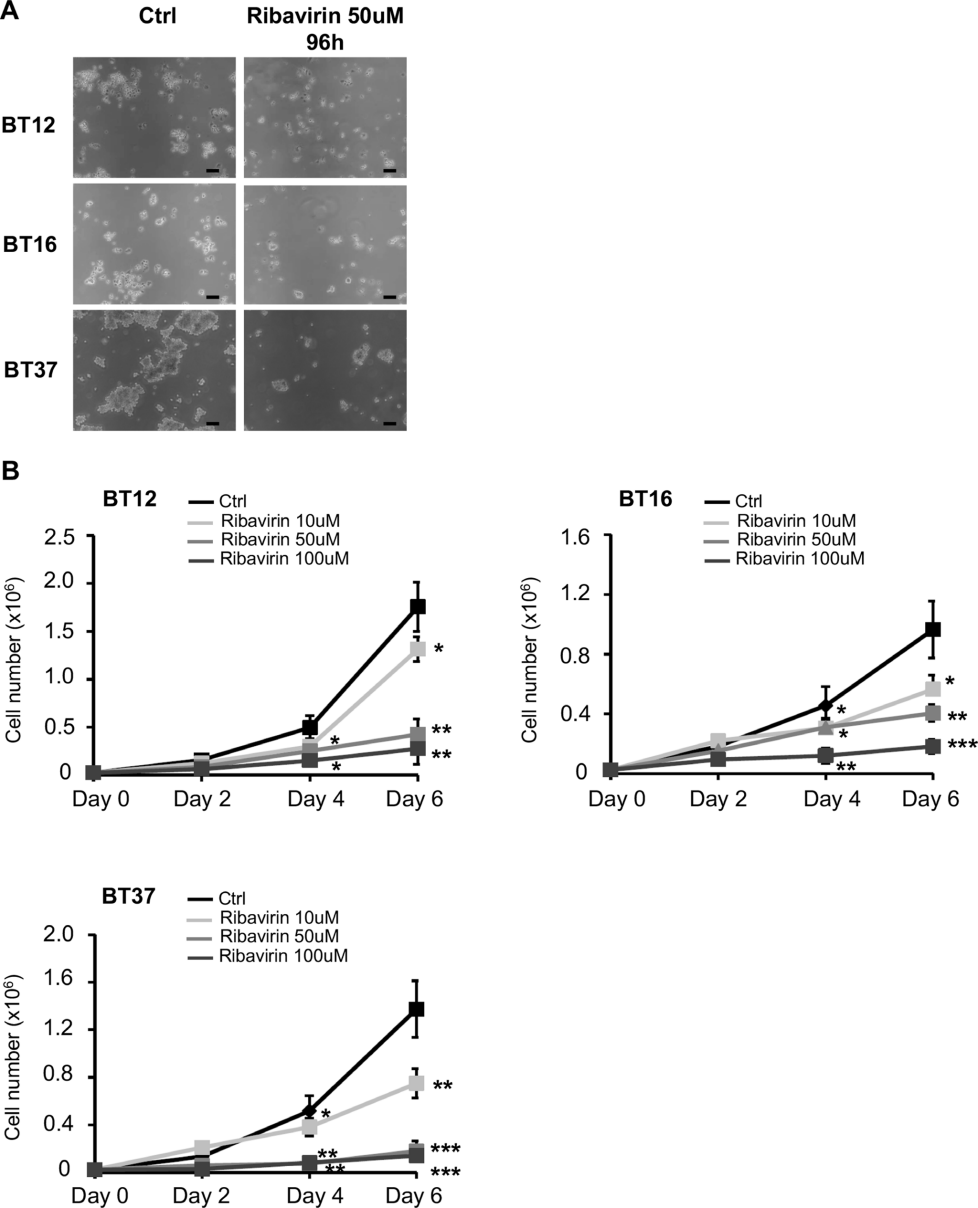
Ribavirin inhibits AT/RT cell proliferation (**A**) Representative images of BT12, BT16, and BT37 cells treated with ribavirin (50 μM) or control for 96hrs. Scale bar=30μm (**B**) Proliferation assays performed with BT12, BT16, and BT37 cells show a decreased cell number in presence of ribavirin, particularly at 50 μM and 100 μM (black curve, Ctrl: PBS vehicle control; light grey curve, Ribavirin: 10 μM; medium grey curve, Ribavirin: 50 μM; dark grey curve, Ribavirin: 100 μM) (^*^p < 0.05, ^**^p < 0.01, ^***^p < 0.001 Ribavirin vs. Ctrl, *n* = 3).

Studies carried out by our laboratory and others previously demonstrated that ribavirin affects viability and cell growth of several cancer cell lines [10–14, 17, 20, 21]. More precisely, in various glioma cell lines, studies have established that the IC50 of ribavirin ranged between 10 μM and 100 μM [21]. In the present work, we specifically selected three different human AT/RT cell lines (BT12, BT16, and BT37) based on their common use and applicability in the AT/RT field as well as their significant differences in terms of AT/RT heterogeneity (Supplementary Figure 1A). We first assessed the effect of ribavirin on tumor cell growth. We treated these AT/RT cells with 10 μM, 50 μM, or 100 μM of ribavirin and subsequently followed cell viability, represented by the number of viable cells, over 6 days (Figure 1A and 1B). As shown in Figure 1A and 1B, ribavirin reduced AT/RT cell growth, and this was particularly significant at day 4 (96 hrs) after treatment. Furthermore, we observed that ribavirin treatment, as low as 10 μM, leads to a significant decrease in AT/RT cell growth (Figure 1B). In order to exclude any toxicity due to indirect effects, such as acidification of the media or nutrient exhaustion, we performed the same experiment and replaced the media with fresh media containing ribavirin every day for the time of the proliferation assay (data not shown). Using these conditions, ribavirin's effect on AT/RT cell growth was preserved, suggesting that ribavirin exerts a targeted effect on AT/RT cells. Taken together, these data show that ribavirin inhibits AT/RT cell growth *in vitro*.

**Figure 2:**
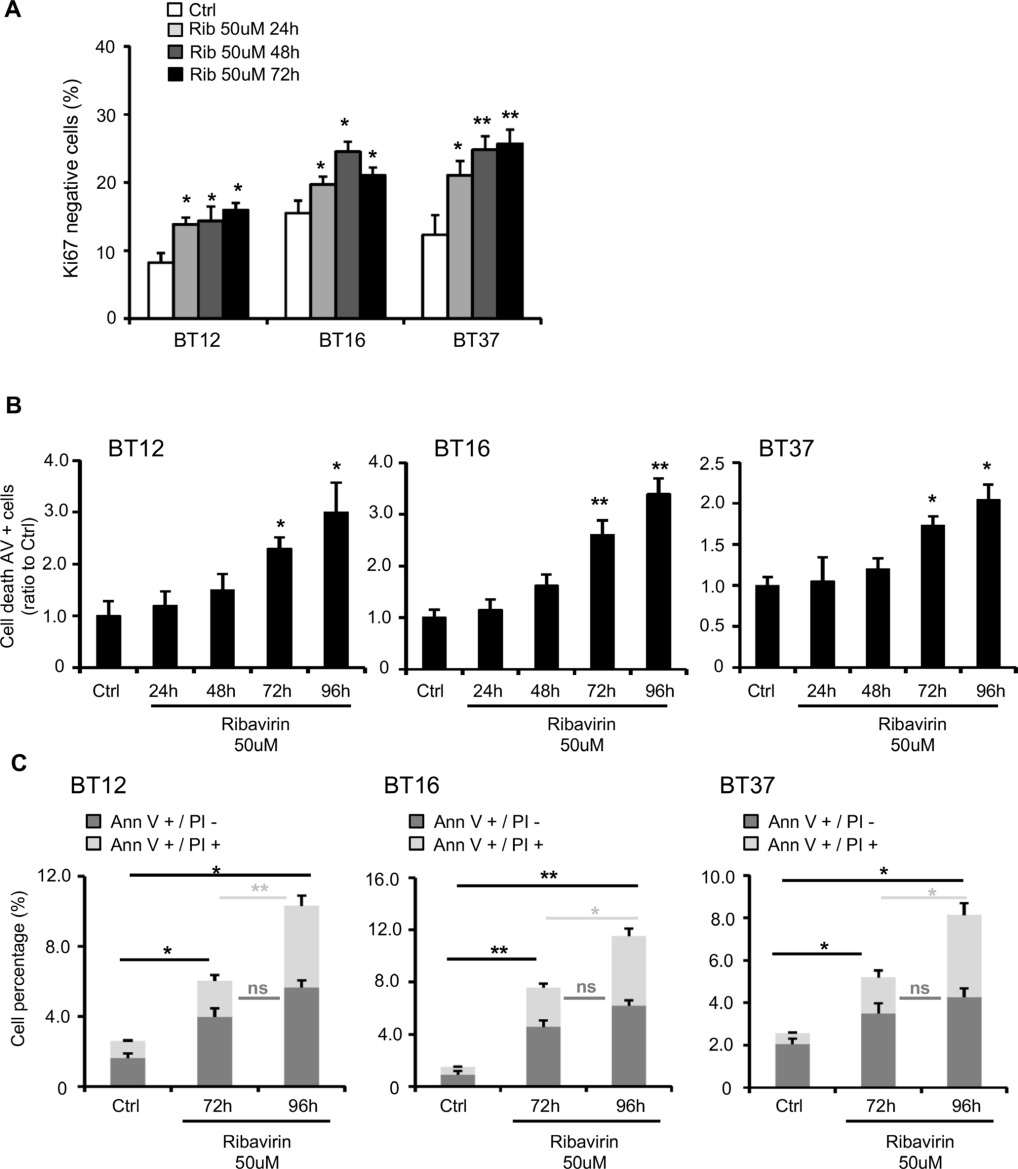
Ribavirin impairs AT/RT cell cycle and induces cell death (**A**) Assessment of Ki67-negative AT/RT cells, using Ki67/PI (Propidium Iodide) staining, 24, 48, and 72hrs after ribavirin treatment. Ribavirin significantly increases the number of arrested cells (^*^p < 0.05, ^**^p < 0.01 Ribavirin compared to Ctrl, *n* = 3). (**B**). Quantification of cell death for BT12, BT16, and BT37 cells using flow cytometry and Annexin-V (AnnV)/PI staining, 24, 48, 72 and 96hrs after treatment. Ribavirin (50μM) significantly increases the number of AnnV+ cells (^*^p < 0.05, ^**^p < 0.01, Ribavirin compared to control, *n* = 3). (**C**) Detailed percentages of AnnV+/PI- and AnnV+/PI+ cells after 72 and 96hrs of ribavirin treatment. Ribavirin (50μM) significantly increases the number of AnnV+/PI- and AnnV+/PI+ cells (ns: non-significant, ^*^p < 0.05, ^**^p < 0.01, *n* = 3).

To explain the effect of ribavirin on AT/RT cell growth, we first assessed cell cycle changes in AT/RT cells treated with either ribavirin or vehicle control through time course experiments. Using flow cytometry and staining for Ki-67, a marker of cell proliferation, we determined the fraction of cells arrested in the G_0_ phase [22–24]. Ribavirin treatment induced a significant increase in the number of Ki67-negative cells starting at 24 hrs after treatment in BT12 (Ribavirin: 13.84% ± 1.02 of Ki67-negative cells vs Ctrl: 8.2% ± 1.44), BT16 (Ribavirin: 19.67% ± 1.18 of Ki67-negative cells vs Ctrl: 15.50% ± 1.74), and BT37 cells (Ribavirin: 21.05% ± 2.1 of Ki67-negative cells vs Ctrl: 12.3% ± 2.88) (Figure 2A). The number of cells in G_0_ continues increasing throughout the time course of the experiment in the presence of ribavirin to reach 15.94% ± 3.21 for BT12, 21.04% ± 1.85 for BT16 and 25.7% ± 3.07 for BT37 at 72 hrs (Figure 2A). Additionally, it is known that cell death and apoptosis can occur in response to cell cycle arrest [22] and we also previously demonstrated that ribavirin induces apoptosis in human and murine glioma and human GBM stem-like cells [10]. Using flow cytometry and Annexin-V/propidium iodide (PI) staining, we assessed ribavirin's effect on AT/RT cell death at 24, 48, 72, and 96 hrs after ribavirin treatment (Figure 2B and 2C). We observed that the apoptotic cell death rate was significantly increased in BT12 (2.3 fold), BT16 (2.6 fold), and BT37 cells (1.73 fold) in response to 72 hr-ribavirin treatment compared to the vehicle-treated cells (Figure 2B and Supplementary Figure 1B). Of note, differentiating between Annexin-V-positive/PI-negative (early apoptosis) and Annexin-V-positive/PI-positive (late apoptosis) cell populations (Figure 2C), we observed that the percentage of Annexin-V-positive/PI-negative cells were comparable at 72 hrs and 96 hrs following ribavirin treatment. However, the percentage of Annexin-V-positive/PI-positive cells were significantly augmented at 96 hrs compared to 72 hrs after treatment, suggesting that cells are transitioning from an early apoptotic stage to a late apoptotic stage over time. These time course experiments allowed us to clarify the timeline of the different processes occurring in response to ribavirin treatment. More specifically, we were first able to detect cell cycle arrest as early as 24 hrs after ribavirin treatment, reflected by the increased percentage of Ki67-negative cells (Figure 2A). Cell death was then consistently observed at 72 hrs and particularly 96 hrs following ribavirin treatment (Figure 2B–2C). Taken together, these findings strongly suggest that ribavirin inhibits human AT/RT cell proliferation through induction of cell cycle arrest, which would precede cell death processes.

### Ribavirin impairs AT/RT cell migration, invasion, and adhesion capacities

**Figure 3:**
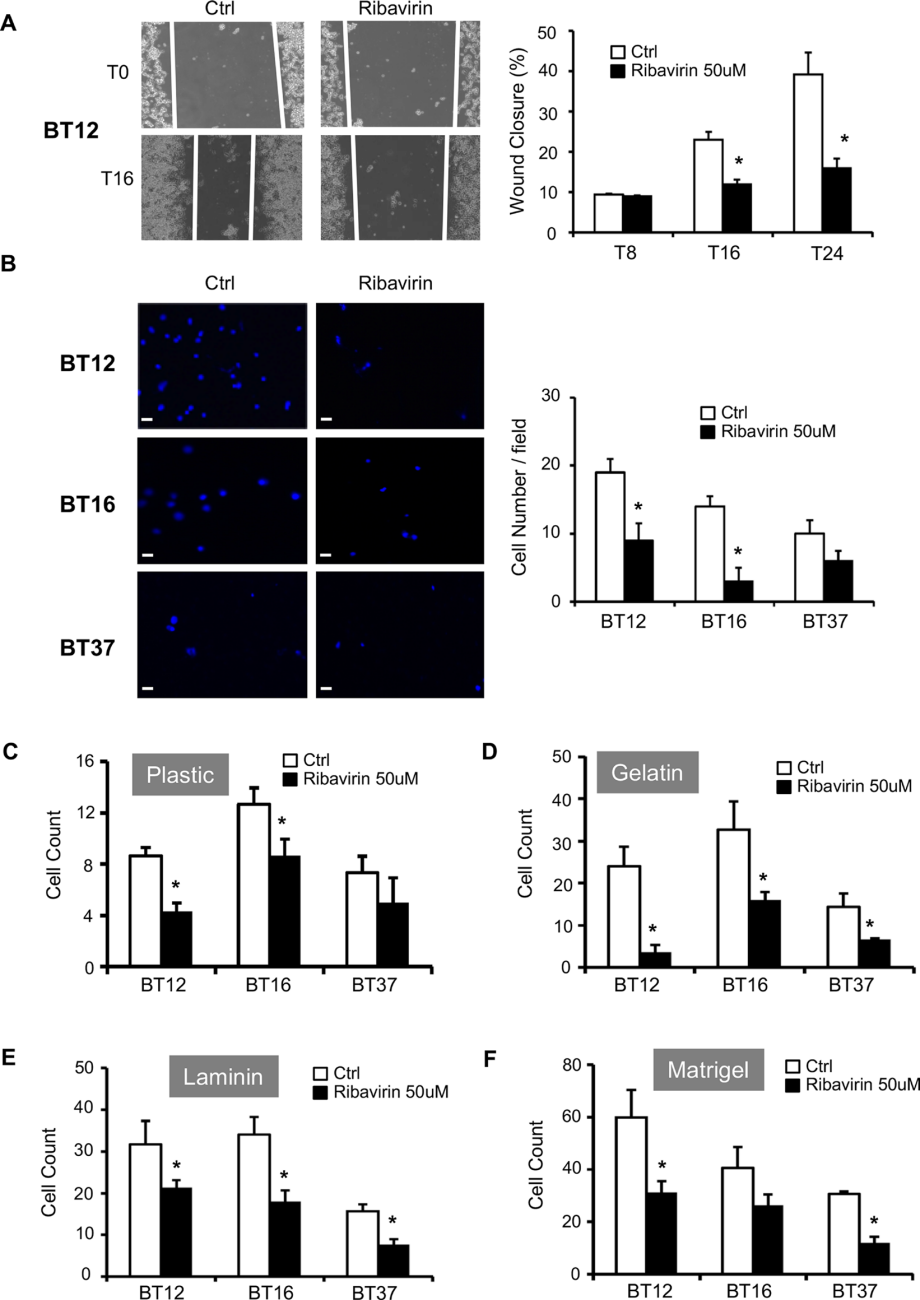
Ribavirin reduces AT/RT migratory, invasive, and adhesive capacities (**A**) Migration of control BT12 cells (Ctrl) and ribavirin-treated cells (Ribavirin 50μM) was assessed using scratch wound assays. Representative photographs were taken immediately after the scratch (T0), and 16hrs later (T16). Quantifications of the percentage of wound closure determined at 8hrs (T8), 16hrs (T16), and 24hrs (T24) after scratch. Wound closure was significantly decreased for ribavirin-treated cells compared to vehicle-treated control cells at 16hrs and 24hrs (^*^p < 0.05, *n =* 3). (**B**) Invasion of control cells (Ctrl) and ribavirin-treated cells (50μM) was assessed using a Boyden chamber assay. Representative photographs were taken after 16hrs. Scale bar=15μm. Quantification of the number of cells per field (at least 6 fields) was determined at 16hrs. Invading cell number was significantly decreased for BT12 and BT16 ribavirin-treated cells compared to vehicle-treated control cells at 16hrs (^*^p < 0.05, *n =* 3). Adhesion capacities of BT12, BT16, and BT37 cells treated or not with ribavirin (50μM) on plastic (**C**), gelatin (**D**), laminin (**E**) and Matrigel^TM^ (**F**) were determined using adhesion assays. Ribavirin treatment significantly decreased the number of adherent cells on each substrate (^*^p < 0.05, ribavirin compared to control, *n* = 3).

Strong invasive and migratory properties are a key feature of AT/RT cells [3]. Based on previous studies conducted on breast cancer and glioma cells showing that ribavirin impairs their invasive capacities [10, 12], we first decided to assess BT12 cell migration in a scratch wound assay (Figure 3A). Of note, BT16 and BT37 do not form an adherent and consistent monolayer of cells, preventing us from performing this assay on these cells. Interestingly, ribavirin treatment significantly decreases BT12 cell ability to close the wound compared to control cells, particularly at 16 hrs and 24 hrs after the scratch (Figure 3A). We also performed a transwell Boyden chamber assay to evaluate BT12, BT16, and BT37 cell invasion and migratory capacities (Figure 3B and Supplementary Figure 1C). As shown in Figure 3B, both BT12 and BT16 cells treated with ribavirin exhibit decreased invasion properties compared to control cells.

In parallel with the decreased migratory and invasive properties of AT/RT cells treated with ribavirin, we studied the effect of ribavirin on AT/RT cell adhesion using plastic, gelatin, laminin, and Matrigel™ substrates (Figure 3C–3F). Ribavirin significantly impaired BT12 cell adhesion on all four substrates (Figure 3C–3F). BT16 cell adhesion was significantly impaired on plastic, gelatin, and laminin (Figure 3C–3E), while BT37 cell adhesion was significantly impaired on gelatin, laminin, and Matrigel™ (Figure 3D–3F). Ultimately, these data show that ribavirin significantly impairs AT/RT cell adhesion on a majority of substrates. These results, combined with those obtained on AT/RT cell migration and invasion, demonstrate that ribavirin could represent an effective therapy to block AT/RT invasive capacities.

### Potential mechanisms of ribavirin action in AT/RT cells

**Figure 4:**
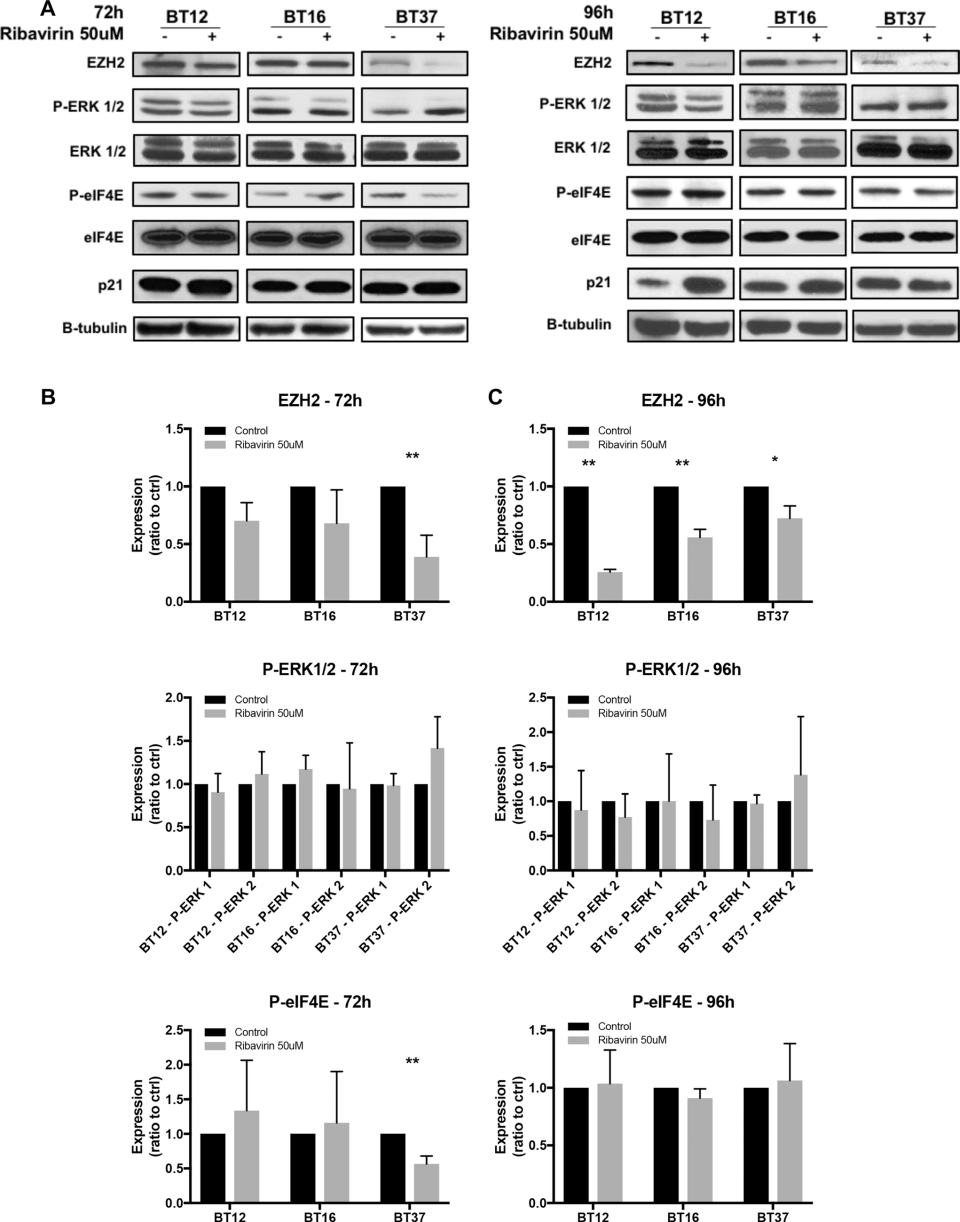
Potential mechanisms of action of ribavirin in AT/RT cells (**A**) Western blot analyses of EZH2, ERK1/2, eIF4E, and p21 expression in BT12, BT16, and BT37 cells 72 and 96hrs after ribavirin treatment. (**B**–**C**) Quantifications of Western blot analyses using Image J software (^*^p < 0.05, ^**^p < 0.01 compared to control, *n* = 3). Ribavirin treatment results in decreased EZH2 expression, decreased eIF4E phosphorylation, and increased p21 expression in a cell line-specific and time-dependent manner.

We next sought to investigate the potential mechanisms involved in response to ribavirin treatment in AT/RT cells (Figure 4, Supplementary Figure 2A). Various studies have documented ribavirin acting via several pathways in cancer cells, including the IMPDH [16, 17], ERK/MAPK [14], eIF4E [15, 20], and EZH2 [17] pathways. Interestingly, EZH2 was recently shown to be one of three genes highly expressed in almost all samples in a genetic and epigenetic study of 192 AT/RTs. As EZH2 is a known target of ribavirin, and EZH2 levels are known to be elevated in AT/RT, we assessed EZH2 expression in our cells after 72 hrs and 96 hrs of ribavirin treatment. Interestingly, EZH2 expression was significantly decreased in BT37 cells at 72 hrs (Figure 4B), and in all three tested cell lines at 96 hrs (Figure 4C). We further demonstrated dose dependency of this ribavirin-induced decrease in EZH2 expression in BT12 cells, as BT12 cells appeared to be the most sensitive at 96 hrs (Supplementary Figure 2A). In addition, we analyzed levels of eIF4E and phospho-eIF4E, which are known to play a pivotal role in protein synthesis and are upregulated in many malignancies [25]. Total eIF4E expression (Figure 4A–4C) was unchanged in all cell lines, while BT37 cells exhibited a consistent decrease in eIF4E phosphorylation (S209) at 72 hrs. Furthermore, eIF4E subcellular localization was also unchanged in BT12 and BT16 cells (Supplementary Figure 2B). Finally, we looked at the ERK/MAPK and p21 proteins (Figure 4A–4C). ERK1/2 phosphorylation levels were variable among cell lines and demonstrated no significant changes in response to ribavirin treatment. Of note, BT37 cells overall exhibited lower levels of phosphorylated ERK-1. Interestingly, in response to ribavirin treatment all three cell lines showed increased expression of the tumor suppressor p21 (concurrently at 72 hrs; and in both BT12 and BT16 at 96 hrs), which is implicated in growth arrest, correlating well with our proliferation (viability) and flow cytometry (Ki67 and Annexin-V/PI) findings.

### Ribavirin significantly improves survival of mice orthotopically implanted with BT12 cells

**Figure 5:**
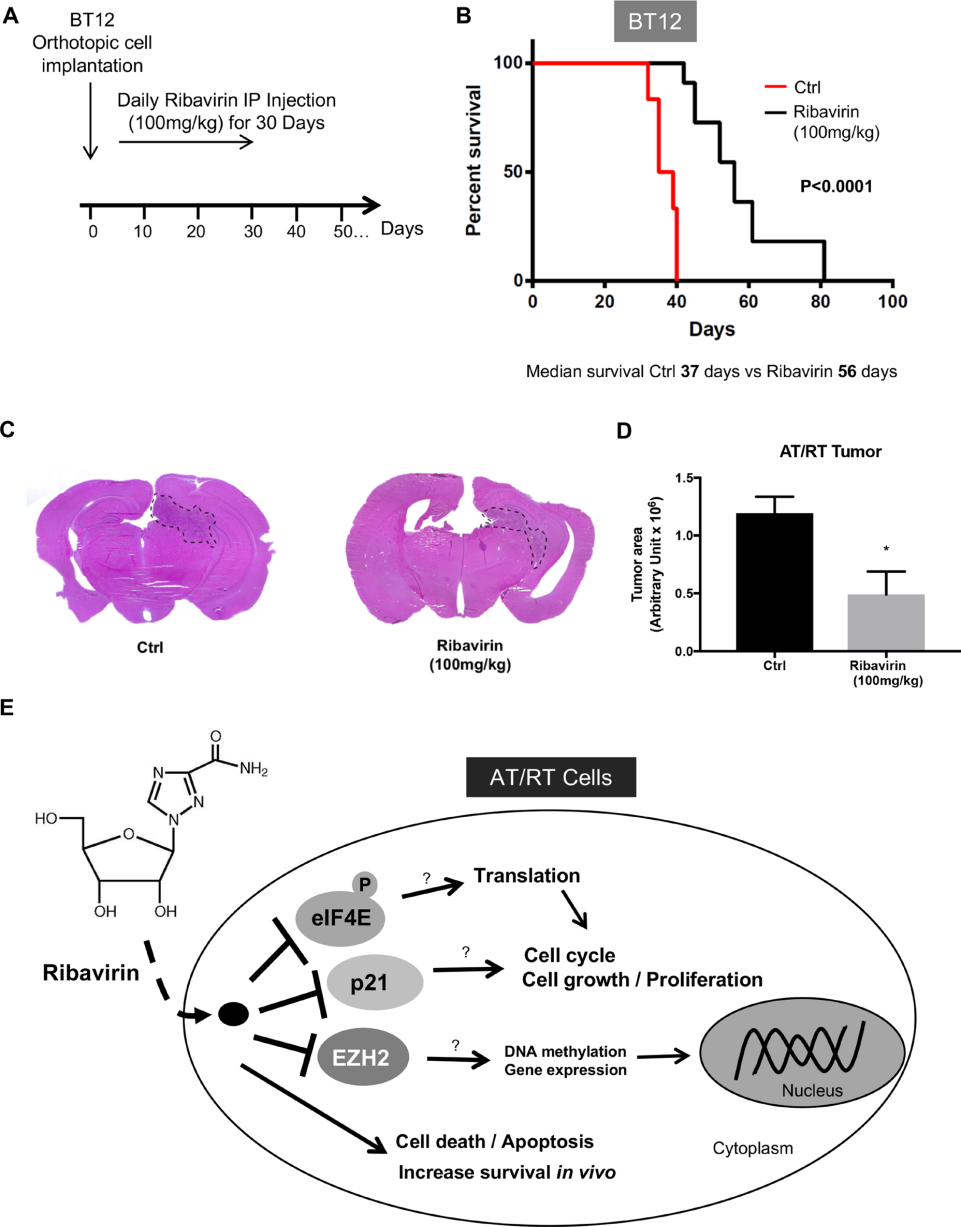
Increased survival of ribavirin-treated mice intracranially implanted with BT12 cells (**A**) After orthotopic BT12 cell implantation, mice were treated daily with IP injection of ribavirin 100mg/kg or vehicle control. (**B**) Kaplan-Meier survival curves following intracranial implantation of BT12 cells in immuno-deficient *(Nu/Nu)* mice and treatment with vehicle control (red curve, *n* = 6) or ribavirin (100mg/kg, black curve, *n* = 6). Ribavirin-treated animals exhibit a significantly increased median survival (56 days) compared to controls (37 days) (*p* < 0.0001). (**C**) Representative photographs of H&E stained sections of mouse brains 20 days after BT12 cell implantation and ribavirin (100 mg/kg) or vehicle treatment. (**D**) Quantification of tumor area revealing significantly decreased tumor size at day 20 after implantation in the ribavirin-treated group (*p* < 0.05). (**E**) Schematic representing the effects of ribavirin treatment on AT/RT cells. Ribavirin impairs multiple components of AT/RT tumorigenicity including cell proliferation, cell cycle progression, cell survival, and migration/invasion and adhesion properties. *In vivo,* ribavirin improves survival in an orthotopic model of AT/RT.

Recent studies have demonstrated that ribavirin was successful at delaying tumor growth *in vivo* in several cancer types, and we also recently demonstrated that ribavirin treatment led to increased survival in two different *in vivo* glioma models [10]. Given this background and the promising results of ribavirin's effects on AT/RT *in vitro*, we next sought to investigate for the first time the effects of ribavirin on AT/RT *in vivo*. We subsequently implanted human AT/RT BT12 cells into the brains of immuno-compromised mice and assessed survival with or without ribavirin treatment. As shown in Figure 5A, mice were intraperitoneally injected daily for 30 days with either vehicle (H_2_O) or 100 mg/kg ribavirin. Very excitingly, ribavirin offered an anti-neoplastic effect *in vivo* as mice treated with ribavirin demonstrated a highly significant increase in survival compared to vehicle-treated controls (Figure 5B). For the control group implanted with human AT/RT BT12 cells, the median survival was 37 days, while with ribavirin treatment (100 mg/kg) this median survival was significantly (*p* < 0.0001) extended to 56 days (Figure 5B). In addition, we harvested mouse brains from vehicle control and ribavirin-treated groups at day 20 after implantation to perform H&E staining and assess tumor size. Images (Figure 5C) and quantifications of tumor area (Figure 5D) revealed that AT/RT tumors were significantly smaller in the ribavirin-treated group compared to the respective controls at day 20, which aligns with the extended median survival. These results demonstrate that ribavirin affects AT/RT growth *in vivo* and represents a promising therapeutic approach in the treatment of AT/RT.

## DISCUSSION

AT/RT are known to be highly aggressive, malignant tumors, and the most common malignant brain tumors in children under 6 months of age, yet there is no current standard of treatment [2, 3]. It is therefore imperative for the scientific and medical communities to identify and develop new and innovative therapeutics for this disease. The synthetic guanosine analogue, ribavirin, is a drug well known for its anti-viral properties and has been used to treat hepatitis C, influenza, parainfluenza, herpes, Lassa fever, measles, chicken pox, and RSV [5, 6]. Recent findings, particularly those observed *in vivo,* have demonstrated that ribavirin also has an unexpected role as a potential anti-tumor therapeutic [8–12, 20].

In the present study, we show for the first time that ribavirin significantly decreases cell growth in all AT/RT cell lines tested (Figure 1). These findings corroborate previous works demonstrating that ribavirin decreased cell growth in various cancer cells, including leukemia [8], breast cancer [12, 13] and glioma cells [10, 16, 21]. We were also able to demonstrate that the decreased AT/RT cell growth could potentially be explained by increased cell cycle arrest, represented by a decrease in the number of AT/RT cells undergoing cell division. Interestingly, these observations also correlate with studies showing ribavirin inducing a G_1_ to G_0_ phase transition in melanoma [20, 26] and glioma cells [10]. As we previously reported for GBM cells, we show here that, in addition to inducing cell cycle arrest, ribavirin induces AT/RT apoptosis, as reflected by the increased percentage of Annexin-V positive cells (as well as a marked transition from Annexin-V positive/PI negative to Annexin-V positive/PI positive staining). Time course experiments of our AT/RT cells following ribavirin treatment clarified the timeline of the different processes induced in response to ribavirin. Our data suggest that ribavirin first induces cell cycle arrest followed by cell death, both of which could ultimately contribute to the observed reduction in AT/RT growth (Figures 1 and 2).

Mechanistically, our data show that ribavirin modulates EZH2, eIF4E, and p21 expression in AT/RT cells in a cell line-specific and time-dependent manner (Figure 5E). These findings corroborate previous studies which demonstrate ribavirin to target key players involved in transcription, translation, and tumorigenesis. Ribavirin has been shown to modulate the onco-protein eIF4E, which plays a pivotal role in protein translation, as well as the histone methyltransferase EZH2. These pathways are well-established ribavirin targets and have been shown to play critical roles in brain tumor cellular proliferation, tumorigenesis or death. Indeed, Kentsis et al. recently demonstrated that ribavirin inhibits 7-methylguanosine (m^7^G) mRNA cap binding to eIF4E [15, 20]. Interestingly, eIF4E over-expression has been established in other CNS malignancies such as GBM [27, 28], while ribavirin treatment of GBM cells has been shown to decrease phosphorylation of eIF4E [10], suggesting a potential target in CNS tumor cells. Furthermore, AT/RT are defined histologically and molecularly by loss of *SMARCB1 (BAF47/INI1/SNF5)* [29–32] and its gene product, SNF5, which is a core component of the SWI/SNF chromatin remodeling complex [18, 33]. This complex and its core SNF5 protein mediate p53 expression and ultimately cell survival, via eIF4E [34]. Studies showed that knockdown of SNF5 led to decreased expression of eIF4E and decreased translation of p53, while restoration of eIF4E resulted in restoration of p53 as well as cell survival [34]. Other studies have revealed over-expression of *ErbB2* and *ErbB3* in AT/RT as well as downstream activation of the Ras/Raf/MEK/ERK pathway [35, 36], providing an additional potential target of ribavirin. Finally, EZH2 has been shown to have a role in transcriptional repression through H3K27 tri-methylation and is considered a key potential target in many cancers [19]. In a recent genetic and epigenetic analysis of 192 AT/RT samples, components of the Polycomb Repressive Complex 2 (PRC2) - *EZH2*, *SUZ12,* and *EED*, were genes found to be highly expressed in a majority of specimens [18, 37]. This implies some antagonistic effects between members of the PRC2-SWI/SNF complexes for regulating epigenetic and chromatin remodeling, but more importantly, this also presents an exciting additional potential target of ribavirin in AT/RT. In fact, recent works have demonstrated that inhibition of EZH2, both genetically and pharmacologically, leads to suppression of cell growth, increased apoptosis, inhibition of tumor sphere formation and radiosensitization in AT/RT cell lines [19]. Additionally, pharmacologic treatment in an *in vivo* xenograft AT/RT model resulted in dose dependent regression of tumors [38]. Interestingly, our data show that the effects on EZH2 are consistently observed in three different AT/RT cell lines (though time dependent) and exhibit dose dependence in the BT12 cell line. EZH2 was consistently targeted by ribavirin in all the cell lines. This positions EZH2 as a target in the cell lines tested, though important to note the significant changes observed in other pathways, in a cell line-specific and time-dependent manner. These effects of ribavirin on multiple critical pathways in AT/RT cells, likely present an advantage in preventing resistance to treatment. However, additional studies are required to further delineate the mechanisms of action of ribavirin on these pathways and the relative contributions of each pathway to the cellular response to treatment.

Finally, in order to confirm *in vivo* the anti-neoplastic effect of ribavirin observed *in vitro*, we assessed for the first time the effect of ribavirin in an orthotopic mouse model of AT/RT. Mice were orthotopically implanted with BT12 cells and we demonstrated a significant extension of the median survival of the ribavirin-treated group compared to the control group by 19 days (*p* < 0.0001). Importantly, since ribavirin is one of the most commonly used drugs to treat hepatitis C, the relevant pharmacokinetic and dosing studies in mice, rats and humans (child and adult) are well documented [12, 39–44]. In repurposing ribavirin as an anti-cancer therapeutic strategy, particularly for breast and cervical cancer, studies with mice utilize ribavirin doses between 50 mg/kg/day and 100 mg/kg/day [12, 44]. In breast tumor mouse models, ribavirin at a dose of 75 mg/kg/day inhibits tumor growth and reduces the development of lung metastases [12]. In cervical cancer murine models, 50 mg/kg/day and 100 mg/kg/day also reduced tumor growth [44]. Interestingly, these doses corresponded to a human dose of 250 mg/day and 500 mg/day, respectively, for a 60 kg patient [45]. For our studies, we previously performed a maximum tolerated dose (MTD) study before assessing the *in vivo* efficacy of ribavirin in mice [10]. We determined that 100 mg/kg was a safe and appropriate dose to study the effect of ribavirin *in vivo*. Clinically, adult patients with hepatitis C tolerate doses ranging from 800–1500 mg/day. Additionally, in a Phase 1 clinical trial for patients with acute myeloid leukemia (AML), ribavirin treatment was used in combination with cytarabine at a dose as high as 2200 mg/day [46]. Our dose of 100 mg/kg in mice corresponds to a human dose (based on body surface area) of 12 mg/kg for a 20 kg child [45]. Furthermore, several reports in children have shed light on the safety and efficacy of high dose ribavirin for the treatment of various CNS diseases. One study showed that intravenous ribavirin was tolerated and efficacious for the treatment of subacute sclerosing panencephalitis (SSPE) at doses as high as 30 mg/kg three times per day (equivalent to 90 mg/kg/day). Adverse events reported at the highest dose included moderate reversible anemia (Hgb 9.7 g/dl) and oral mucosal swelling [47]. The authors found that when the dose of ribavirin was increased from 10 mg/kg to 30 mg/kg, serum concentration of ribavirin increased from 1.3 to 20.9μg/ml [47]. This is equivalent to a serum concentration of 85.58 μM, a concentration in the range of our *in vitro* experiments and higher than the 50 μM concentration that is consistently used for all our *in vitro* work. Knowing that the bioavailability of ribavirin through oral administration is almost equal to intraperitoneal or intravenous injection, and that ribavirin is usually prescribed long-term for 24–48 weeks, these ribavirin doses can be achieved through oral administration. Additionally, these doses are safe, as children with hepatitis C are known to tolerate doses of 30–400 mg/day. Moreover, it is well documented that ribavirin penetrates well into the CSF of patients with SSPE and HIV, achieving 74% (ranging 50–89%) of the concentration in the serum [47, 48]. This leads us to believe that in a shorter period of time, higher concentrations could be achieved in order to maximize the inhibitory effects of ribavirin on AT/RT cell growth.

In conclusion, AT/RT research is an area of unmet need, standard treatments do not exist, and current treatments fail to cure. Here, we report that ribavirin has the ability to reduce AT/RT cell tumorigenesis *in vitro* and more importantly, *in vivo* (Figure 5). Ribavirin is currently being investigated in numerous clinical trials for its anti-neoplastic activity in various cancers, particularly acute myeloid leukemia (NCT02109744, NCT02073838) [46], head and neck cancer (NCT01268579) and metastatic breast cancer (NCT01056757). Our laboratory and others are also investigating the benefits of combining ribavirin with chemotherapies and radiotherapy [11, 49]. This is of crucial importance, as we and others have highlighted that ribavirin could enhance the cytotoxic effects of radio- and chemotherapy in GBM and breast cancer models. Ultimately, given the need to introduce new forms of treatment for children with AT/RT, we believe that our results and the fact that ribavirin is already FDA approved should allow us to move forward and evaluate ribavirin efficacy in a clinical setting in the near future.

## MATERIALS AND METHODS

### Cell lines and culture conditions

Human AT/RT cell lines, BT12, BT16, and BT37 (obtained from C. Eberhart's laboratory, Johns Hopkins University, Baltimore, MD, USA) were used and routinely maintained in Dulbecco's Modified Eagle Medium or RPMI medium (Lonza, Portsmouth, NH, USA) supplemented with 10% fetal bovine serum (Lonza) at 37°C in 5% CO_2_-humidified incubators as previously described [10, 24, 50, 51]. When indicated, cells were treated with the following compounds: Phosphate Buffered Saline (PBS) or ultrapure water as vehicle, 10μM, 50μM and 100μM ribavirin (1-β-D-ribofuranosy-1,2,4-triazole-3-carboxamide, Sigma-Aldrich, St Louis, MO, USA).

### Cell growth–viability assay

When indicated, AT/RT cells (2.5 × 10^4^) were treated with 10-50-100 μM ribavirin (Sigma-Aldrich) or Phosphate Buffered Saline (PBS, Lonza) as vehicle. Cells were then collected and counted using the Malassez slide (Invitrogen, Carlsbad, CA, USA).

### Flow cytometry

At 24, 48, 72, or 96 hrs after treatment with ribavirin (50 μM), cells were collected, washed in PBS and prepared for flow cytometry. For cell death, we used reagents from the APC-AnnexinV/Dead Cell Apoptosis Kit (Invitrogen). For cell cycle analysis, cells were labeled with FITC-anti-Ki67 antibody (Abcam, Cambridge, MA, USA) and run on a flow cytometer (FACS Calibur, Becton-Dickinson, Franklin Lakes, NJ, USA). Subsequent analyses were performed using FlowJo software (FlowJo LLC, Ashland, OR, USA).

### Migration / adhesion assay

Migration and Adhesion assays were performed as described previously [52]. For the adhesion assay, ribavirin-pretreated (48 hrs) or control cells were seeded into 24-well plates coated with gelatin (2 mg/ml, Sigma-Aldrich), Matrigel™ (10 mg/ml, Corning, NY, USA), or laminin (10 μg/ml, Sigma-Aldrich) and incubated for 1hr at 37°C. Cells were then carefully washed 3 times with PBS, fixed, and counted. The number of remaining adherent cells was counted in at least 4 different wells for each condition using a Zeiss Observer Z.1 AX10 microscope (Zeiss, Thornwood, NY, USA) and ImageJ software (Windows 1.47, Research Services Branch, NIH, Bethesda, MD, USA).

### Invasion assay

The invasion assay was performed using 24-well plates with Matrigel-coated inserts (8μm pores, BD Biosciences). Ribavirin-pretreated and control cells (1×10^5^) were placed in the top chamber containing medium without serum. The lower chamber contained medium with 10% fetal bovine serum. After invasion, non-invasive cells were removed from the top of the insert using cotton swabs. The underside of each membrane was fixed and stained with DAPI (Invitrogen). The number of cells was counted for each condition using a Zeiss Observer Z.1 AX10 microscope (Zeiss). The data are representative of three independent experiments.

### Western blot analysis

Cells treated with ribavirin or control were lysed and Western blots were performed using the following rabbit and mouse antibodies: anti-phospho (S209) eIF4E (1/1000, #9742, Cell Signaling, Beverly, MA, USA), anti-eIF4E (1/1000, #9741, Cell Signaling), anti-EZH2 (1/500, #MA5-18108, ThermoFisher Scientific, Waltham, MA USA), anti-phospho (T202/Y204) ERK (1/1000, #4370, Cell Signaling), anti-ERK (1/1000, #9102, Cell Signaling), and anti-p21 (1/1000, #2947, Cell Signaling). Data were normalized to their respective loading controls using a rabbit anti-β-tubulin antibody (1/1000, #2146, Cell Signaling). Gel quantification was performed using ImageJ software.

### eIF4E subcellular distribution

BT12 and BT16 cells (10-50×10^3^/sample) were plated on microscope slides and treated with ribavirin (50 μM) or control for 72 hrs. Slides were then dried at 37°C, fixed and permeabilized with 100% methanol at −20°C for 20min, washed with PBS, and blocked with 10% fetal bovine serum/0.2% Tween-20/PBS at 37°C for 1hr. Slides were then labeled with 1:50 anti-eIF4E-FITC (BD Transduction) in 10% FBS/0.2% Tween-20/ PBS at 37°C for 2 hrs. Slides were then mounted with Fluoro-Gel II with DAPI (Electron Microscopy Sciences, Hatfield, PA, USA). DAPI and eIF4E images were acquired with a Zeiss Observer Z.1 AX10 microscope (Zeiss).

### Orthotopic xenograft experiments

Mice (NU/NU athymic) were purchased from Charles River (Cambridge, MA, USA) and intracranially implanted with 5×10^5^ BT12 AT/RT cells as previously described [53, 54]. Animals were given intraperitoneal injections (200μL) of pharmaceutical-grade anesthesia, analgesia, and study agents (water vehicle or ribavirin, 100 mg/kg/day). At day 20 after implantation, mouse brains from vehicle- and ribavirin-treated groups were collected, fixed in formalin, paraffin-embedded, sectioned, and H&E stained. Images were taken using a Zeiss Observer Z.1 AX10 microscope (Zeiss), and quantification of tumor sizes was performed using ImageJ software. All procedures were performed in accordance with the guidelines set forth by the Johns Hopkins University Animal Care and Use Committee.

### Statistical analysis

Wilcoxon-Mann-Whitney and unpaired *t*-tests were used to calculate final *p*-values. Data are representative of at least three independent experiments and significance is represented by “^*^” in which ^*^p < 0.05, ^**^p < 0.01, ^***^p < 0.001. For the Kaplan-Meier survival analysis, we compared vehicle-treated and ribavirin-treated groups using the log rank test.

## SUPPLEMENTARY MATERIALS FIGURE


