# Supplementary material for: Ribavirin as a potential therapeutic for atypical teratoid/rhabdoid tumors
Source: Oncotarget. 2018 Jan 3;9(8):8054–67. doi: 10.18632/oncotarget.23883 (PMC5814281; doi:10.18632/oncotarget.23883)
Supplement: Supplementary file 1 [file oncotarget-09-8054-s001.pdf]

Ribavirin as a potential therapeutic for atypical teratoid/rhabdoid tumors

SUPPLEMENTARY MATERIALS

A

Cell line	BT12	BT16	BT37
Primary tumor site	Posterior Fossa	N/A	N/A
Age	2 mo	2 yr	N/A
Sex	Female	Male	N/A
Culture media	DMEM	DMEM	RPMI
Growth properties	Adherent	Adherent	Suspension
MGMT promoter methylation	Unmethylated	Unmethylated	Unmethylated
p53 status	Mutated	N/A	Wild-type
c-MYC expression	High	High	Low
INI1 status	Not expressed	Not expressed	Not expressed
Lin28A expression	Negative	N/A	Positive
Lin28B expression	Positive	N/A	Negative
HMGA2 expression	Negative	N/A	Positive
MITF expression	High	Low	N/A

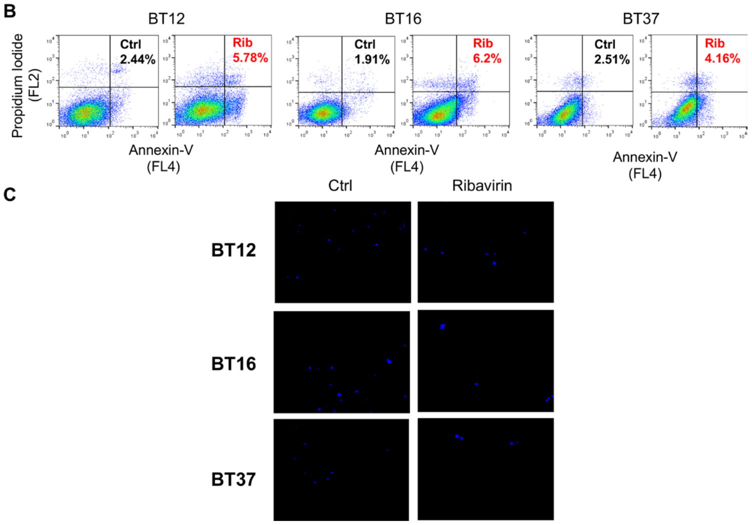

Supplementary Figure 1: AT/RT cell line characteristics and ribavirin effects on AT/RT cell death and invasion. (**A**) Table outlining core features and characteristics of the AT/RT cell lines BT12, BT16, and BT37 used in the study. (**B**) Representative flow cytometry plots of Annexin-V/Propidium Iodide-stained BT12, BT16, and BT37 cells treated with ribavirin (50µM) or vehicle control for 72hrs. (**C**) Additional representative photographs of Boyden chamber assay. Invasion of control cells (Ctrl) and ribavirin-treated cells (50 µM) was assessed using a Boyden chamber assay. Panels show representative photographs taken after 16 hrs. Invading cell number was significantly decreased for BT12 and BT16 ribavirin-treated cells compared to vehicle-treated control cells at 16 hrs ($^{*}p < 0.05$, $n = 3$).

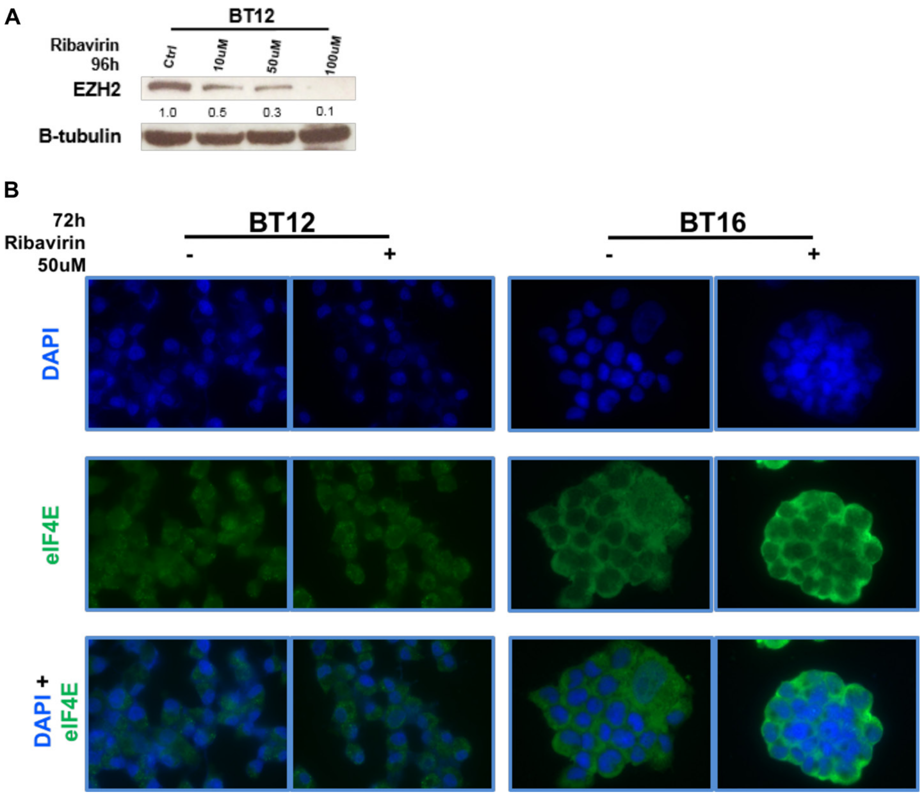

Supplementary Figure 2: EZH2 expression in BT12 cells and eIF4E subcellular localization in BT12 and BT16 cells following ribavirin treatment. (**A**) Western blot analyses of EZH2 expression in BT12 cells after 96hrs of varying doses (10 µM, 50 µM, and 100 µM) of ribavirin treatment. Ribavirin treatment leads to a dose-dependent decrease in EZH2 expression. (**B**) Immunofluorescence experiments for eIF4E were performed to determine its subcellular localization in BT12 and BT16 cells after 72 hrs of treatment with ribavirin (50 µM). Overall, DAPI and eIF4E co-staining reveal no difference in eIF4E localization in ribavirin or vehicle-treated cells. eIF4E was found to be mainly cytoplasmic in both control and ribavirin-treated samples.
